# Identification and Characterization of a Luteinizing Hormone Receptor (LHR) Homolog from the Chinese Mitten Crab *Eriocheir sinensis*

**DOI:** 10.3390/ijms20071736

**Published:** 2019-04-08

**Authors:** Li-Juan Yuan, Chao Peng, Bi-Hai Liu, Jiang-Bin Feng, Gao-Feng Qiu

**Affiliations:** 1 National Demonstration Center for Experimental Fisheries Science Education, Shanghai Ocean University, Shanghai 201306, China; m130111281@st.shou.edu.cn (L.-J.Y.); 15602258587@163.com (C.P.); m170100139@st.shou.edu.cn (B.-H.L.); jbfeng@shou.edu.cn (J.-B.F.); 2 Key Laboratory of Exploration and Utilization of Aquatic Genetic Resources, Ministry of Education, Shanghai Ocean University, Shanghai 201306, China; 3 Key Laboratory of Freshwater Aquatic Genetic Resources, Ministry of Agriculture, Shanghai Ocean University, Shanghai 201306, China; 4 Shanghai Engineering Research Center of Aquaculture, Shanghai Ocean University, Shanghai 201306, China

**Keywords:** luteinizing hormone receptor, expression profile, ovarian development, *Eriocheir sinensis*

## Abstract

Luteinizing hormone (LH), a pituitary gonadotropin, coupled with LH receptor (LHR) is essential for the regulation of the gonadal maturation in vertebrates. Although LH homolog has been detected by immunocytochemical analysis, and its possible role in ovarian maturation was revealed in decapod crustacean, so far there is no molecular evidence for the existence of LHR. In this study, we cloned a novel *LHR* homolog (named *EsLHR*) from the Chinese mitten crab *Eriocheir sinensis*. The complete sequence of the *EsLHR* cDNA was 2775bp, encoding a protein of 924 amino acids, sharing 71% amino acids identity with the ant *Zootermopsis nevadensis* LHR. *EsLHR* expression was found to be high in the ovary, while low in testis, gill, brain, and heart, and no expression in the thoracic ganglion, eye stalk, muscle, and hepatopancreas. Quantitative PCR revealed that the expression level of *EsLHR* mRNA was significantly higher in the ovaries in previtellogenic (Pvt), late vitellogenic (Lvt), and germinal vesicle breakdown (GVBD) stages than that in the vitellogenic (Mvt) and early vitellogenic (Evt) stages (*P* < 0.05), and, the highest and the lowest expression were in Lvt, and Evt, respectively. The strong signal was mainly localized in the ooplasm of Pvt oocyte as detected by in situ hybridization. The crab GnRH homolog can significantly induce the expression of *EsLHR* mRNA at 36 hours post injection in vivo (*P* < 0.01), suggesting that *EsLHR* may be involved in regulating ovarian development through GnRH signaling pathway in the mitten crab.

## 1. Introduction

Luteinizing hormone (LH), a glycoprotein hormone secreted from the pituitary gland, is one of the main factors inducing oocyte maturation and ovulation in vertebrates [1]. Similar with follicle-stimulating hormone (FSH), the release of LH was regulated by gonadotropin-releasing hormone (GnRH) in the hypothalamic-pituitary-gonadal axis (HPG axis) [2]. LH works in coordination with FSH, to induce gonads to synthesize and secrete maturation-inducing hormone (MIH), by combining its specific membrane receptor, LH receptor (LHR). Finally, MIH promotes proliferation of granulosa cell and maturation of germ cell [3].

LHR belongs to the rhodopsin-like G-protein coupled receptor (GPCR) family [4], the largest family of cell-surface receptors [5]. The GPCR family has undergone a long evolutionary history and exacts various biological functions, including animal reproduction [6]. LHR contains a typical structure of GPCR family members with an N-terminal extracellular domain (ECD), a seven transmembrane domain (TMD) and an intracellular domain (ICD) [7,8]. The ECD domain was rich in leucine-rich repeats (LRRs) and contained a highly conserved region of cysteine, which was involved in the hormone-binding process, while the TMD was responsible for signal transduction and G-protein coupling function [9,10].

Due to the important role in the regulation of reproduction, *LHR* has been characterized in a wide variety of vertebrate species, since it was first isolated from human placenta in 1997. In aquaculture species, the expression pattern and function of *LHR* have been examined in many teleost species including *Danio rerio* [11], *Dicentrarchus labrax* [12], *Acanthopagrus schlegeli* [13], *Hippoglossus hippoglossus* [14], *Oncorhynchus rhodurus* [15], *Salmo salar* [16], *Gadus morhua* [17], *Anguilla japonica* [18], *Clarias gariepinus* [19], etc. LHR was primarily expressed in gonads. Additionally, *LHR* exhibited a high expression in head kidney [19,20] and was detectable in other tissues of some fishes [19]. In the gonads, the expression level of the *LHR* exhibits obviously seasonal (reproductive stage-specific) rhythms. Expression of channel catfish (*Letalurus Punetaus*) *LHR* remained rather low at most stages in the reproductive cycle, but was significantly upregulated around the time of spawning [20]. In *D. rerio*, the expression of *LHR* increased along with ovarian development and reached peak level at the full-grown oocyte stage [11]. In decapod crustacean, although LH protein has been detected by immunohistochemical analysis and has implication in ovarian maturation, there is no molecular evidence for the existence of LHR so far. In our previous study, high-throughput next-generation sequencing data analysis of the brain and ovary transcriptomes revealed a putative GnRH signaling pathway in the prawn *Macrobrachium nipponense* [5] and the Chinese mitten crab *Eriocheir sinensis* [21]. Like vertebrates, the expression of the GnRHR homologs has implications in ovarian development of prawns and crabs [5,22]. Therefore, we deduced that LHR could also exist in prawns and crabs, and function as a downstream factor in GnRH pathway in ovarian maturation. To test this hypothesis, in the present investigation we cloned and characterized the mitten crab *E. sinensis LHR* homolog transcript (*EsLHR*). We then examined its tissue distributions and spatial-temporal expression profiles during ovarian development, using quantitative PCR (qPCR) and in situ hybridization (ISH) analysis. We also examined the effect of the crab GnRH homolog on *EsLHR* expression in the ovarian development. The results could help us better understand the molecular mechanism of ovarian maturation and provide useful basic knowledge for the improvement of reproductive manipulation of crabs.

## 2. Results

### 2.1. Molecular Characterization and Phylogenetic Analysis of EsLHR Homolog

**Figure 1:**
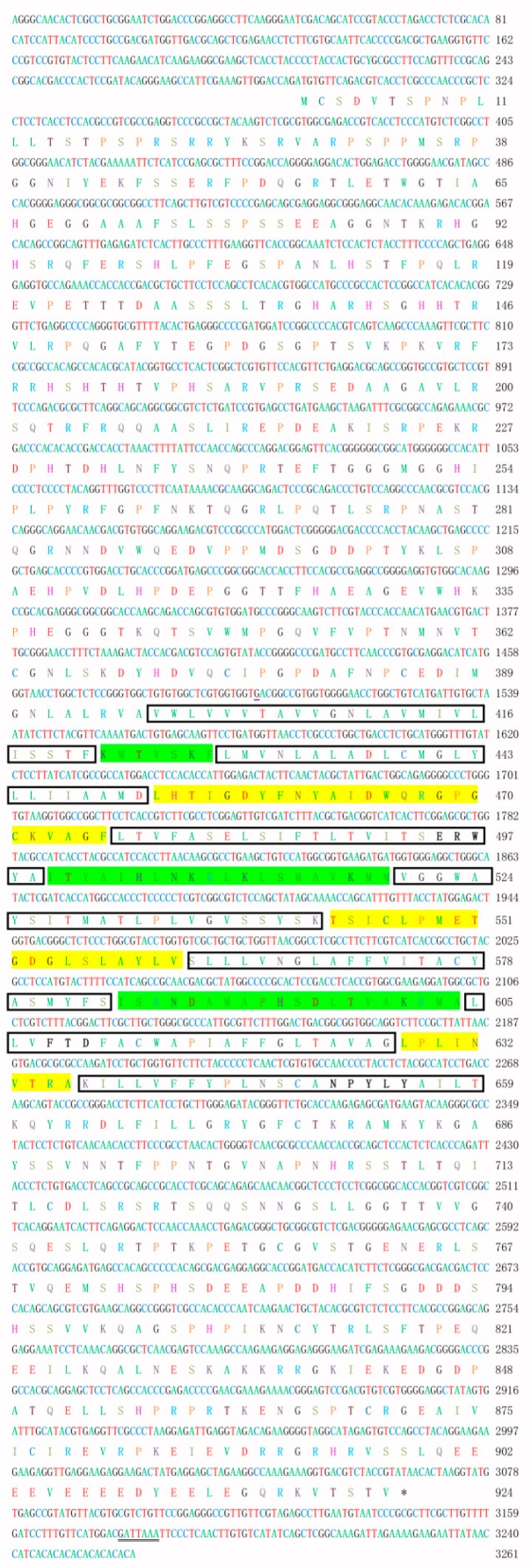
Molecular characteristics of *EsLHR* cDNA and its deduced peptide. The box indicates seven transmembrane helix regions; the green and yellow shadow highlight the intracellular, and the extracellular loop, respectively; the black font represent the special conserved signal sequence of G-protein coupled receptor (GPCR) family; the double underlined ATTAAA indicates a potential polyadenylation signal; the star marks the stop codon.

**Figure 2:**
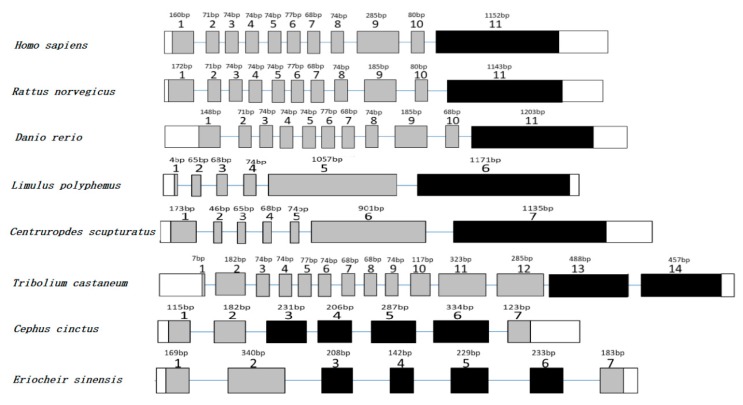
Schematic diagram of *EsLHR* gene structure as compared with various species. The gray and black boxes represent exons, in which the black indicates the exons coding the seven-transmembrane domain. Arabic numerals mark the exon positions. The length of each exon was indicated as base pairs (bp).

**Figure 3:**
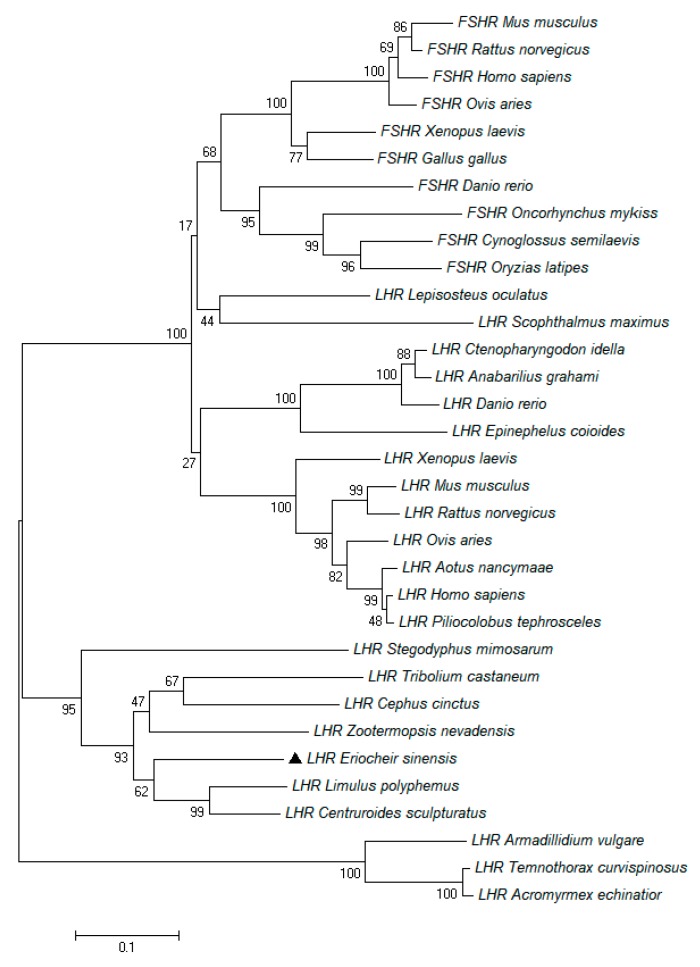
The neighbor-joining phylogenetic tree of EsLHR, and other known G-protein coupled receptor members; alignment of amino acid sequences are produced by Clustal X and MEGA 6.0. The confidence in each node is assessed by 1000 bootstrap replicate. GeneBank accession numbers of above various species: *Lepisosteus oculatus* LHR (XP_015218187.1); *Scophthalmus maximus* LHR (AIE47234.1); *Anabarilius graham* LHR (ROL48811.1); *Aotus nancymaae* LHR (XP_012320900.1); *Pilicolobus tephrosceles* LHR (XP_023079591.2); *Nephila clavipes* LHR (PRD27275.1); *Stegodyphus mimosarum* LHR (KFM82218.1); *Armadillidium vulgare* LHR (RXG67696.1); *Temnothorax curvispinosus* LHR (XP_024869715.1); *Acromyrmex echinatior* LHR (EGI67461.1); *Xenopus laevis* LHR (BAL15689.1); *Homo sapiens* LHR (CAA59234.1); *Ovis aries* LHR (NP_001265495.1); *Mus musculus* LHR (EDL38653.1); *Rattus norvegicus* LHR (NP_037110); *Danio rerio* LHR (AAP33512.1); *Ctenopharyngodon idella* LHR (ABM73668.1); *Oncorhynchus mykiss* LHR (ACD39387.2); *Epinephelus coioides* LHR (AEG65827.1); *Tribolium castaneum isoform* LHR (XP_008195299.1); *Limulus polyphemus* LHR (XP_013780200.1); *Centruroides sculpturatus* LHR (XP_023235072.1); *Cephus cinctus* LHR (XP_024939991.1); *Zootermopsis nevadensis* LHR (KDR08863.1); *Mus musculus* FSHR (EDL38653.1); *Rattus norvegicus* FSHR (EDM02615.1); *Homo sapiens* FSHR (AAI25271.1); *Ovis aries* FSHR (XP_014949269); *Xenopus laevis* FSHR (BAL15688.1); *Gallus gallus* FSHR (NP_990410); *Danio rerio* FSHR (AAP33512.1); *Oncorhynchus mykiss* FSHR (AAQ04551.1); *Orzias latipes* FSHR (BAJ05402.1); *Cynoglossus semilaevis* FSHR (ACD39387.2).

Based on a partial annotated sequence from the ovarian transcriptome library of the *E. sinensis*, the full-length cDNA sequence of the *EsLHR* was amplified by RACE-PCR from the ovary in *E. sinensis* and deposited in GenBank with accession number MK312165. The *EsLHR* cDNA was 2775 bp in length, encoding a putative peptide of 924 amino acids (aa). The 3′-untranslated region (3′-UTR) of the *EsLHR* has a potential polyadenosine with a base sequence of ATTAAA (Figure 1). Like other species, LHR, the deduced EsLHR possess typical features of glycoprotein receptors: N-terminal extracellular domain (ECD), seven transmembrane helix domain (TMD) and C-terminal intracellular domain (ICD), and a highly cysteine-rich region in the glycoprotein hormone receptor family: ERW, FTD and NPFLY (Figure 1). Seven exons were found in *EsLHR* gene after blasted in the genomic dataset of *E. sinensis* (unpublished data). Seven-TMD was encoded by the 3^th^, 4^th^, 5^th^, and 6^th^ exons of *EsLHR* gene (Figure 2). The multiple alignment of the deduced amino-acid sequences of EsLHR, with known LHRs from other species showed that EsLHR shares 52–71% identity with LHR sequences from other species. The highest amino acids identity (71%) was found with the ant *Zootermopsis nevadensis* LHR. To assess the EsLHR protein and its evolutionary relationship, a phylogenetic tree was constructed using the NJ method. The dendrogram shown in Figure 3 depicts the evolutionary relationships based on the sequence similarity of EsLHR proteins from various species. The LHR and FSHR were separated into two clades. EsLHR protein was clustered together with the LHR from the horseshoe crab Limulus polyphemus and the scorpion *Centruroides sculpturatus*, indicating that the EsLHR belongs to LHR family rather than FSHR (Figure 3). 

### 2.2. Tissue Distribution of EsLHR mRNA

**Figure 4:**
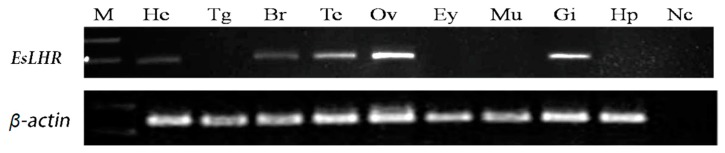
RT-PCR analysis of distribution of *EsLHR* mRNA in different tissues. M: marker; He: heart; Tg: thoracic ganglion; Br: brain; Te: testis; Ov: ovary; Ey: eye stalk; Mu: muscle; Gi: gill; Hp: hepatopancreas; Nc: negative control.

The distribution of *EsLHR* mRNA in brain, ovary, testis, thoracic ganglion, eye stalk, heart, muscle, and hepatopancreas was determined by RT-PCR. As shown in Figure 4, the expression was the highest in the ovary, lower in testis and gill, brain and heart, whereas no expression in the thoracic ganglion, eye stalk, muscle and hepatopancreas. The result indicated that *EsLHR* is transcribed in a tissue-specific manner and may be involve in gonad development.

### 2.3. ISH localization of EsLHR mRNA in the Ovaries at Various Stages

**Figure 5:**
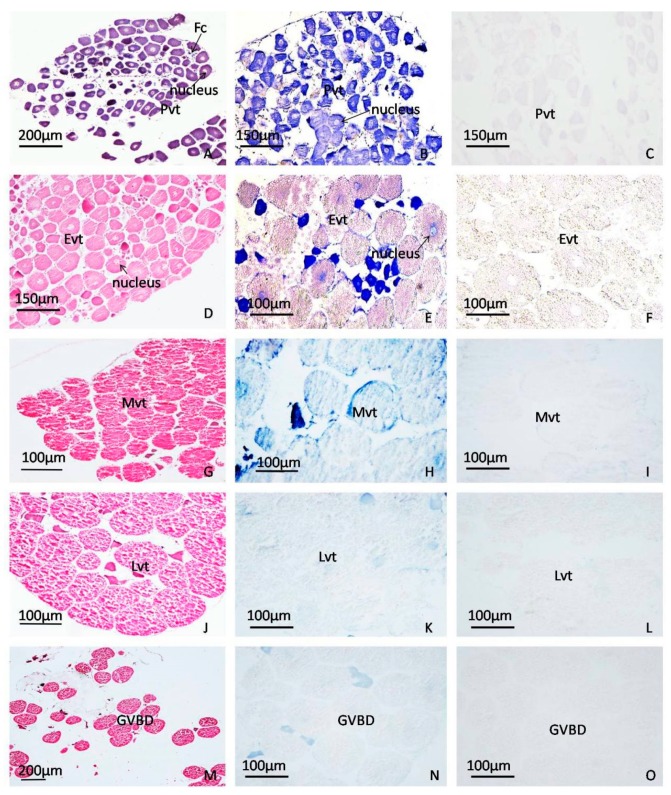
In situ hybridization (ISH) localization of *EsLHR* mRNA in the ovaries at various stages. (**A**–**C**), previtellogenic stage (Pvt); (**D**–**F**), early vitellogenic stage (Evt); (**G**–**I**), middle vitellogenic stage (Mvt); (**J**–**L**), late vitellogenic stage (Lvt); (**M**–**O**), germinal vesicle breakdown stage (GVBD). A, D, G, J, and M, HE staining; B, E, H, K, and N, ISH using antisense probes; C, F, I, L, and O, negative control using sense probes.

Based on the ovarian tissue section observation, crab ovarian development can be classified into previtellogenic (Pvt), early vitellogenic (Evt), vitellogenic (Mvt), late vitellogenic (Lvt), and germinal vesicle breakdown (GVBD) stages. In Pvt, the nucleus of the oocyte was transparent and was termed germinal vesicle (GV), while the ooplasm and nucleolus were notably stained to a dark blue color by hematoxylin (Figure 5A). As appearance of in yolk protein, the ooplasm was stained to red by eosin at Evt, Mvt, Lvt, and GVBD stages (Figure 5D,G,J,M). The ISH results revealed that a strong positive signal was mainly visualized in the ooplasm of Pvt oocytes (Figure 5B). Subsequently, the signal became weaker and weaker as the oocytes grew larger and larger from Evt to Lvt stages (Figure 5E,H,K), and disappeared in the GVBD-oocyte at final maturation stage (Figure 5N). No hybridization signal was detected at any stages of ovarian development when a sense probe was used (Figure 5C,F,I,L,O).

### 2.4. qPCR Quantitation of EsLHR mRNA in the Ovaries at Various Stages

**Figure 6:**
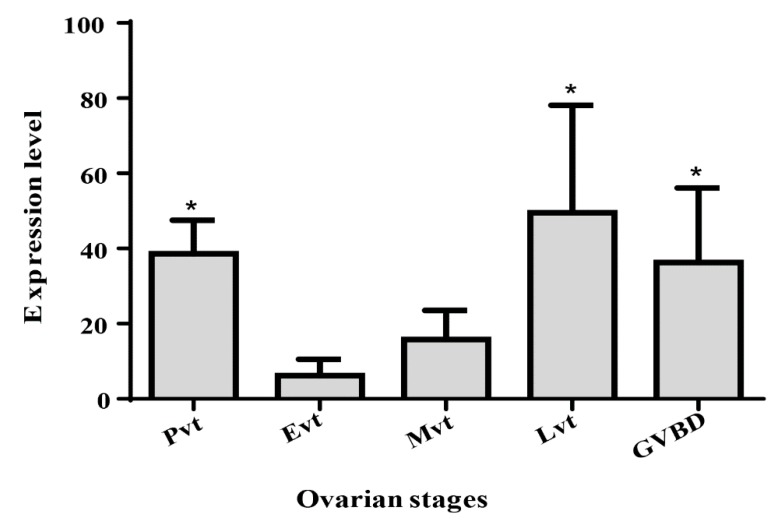
qPCR quantitation of *EsLHR* mRNA expression in the ovaries at various stages. Pvt, previtellogenic stage; Evt, early vitellogenic stage; Mvt, vitellogenic stage; Lvt, late vitellogenic stage; GVBD, germinal vesicle breakdown stage. Vertical bars represented mean ± SE (*n* = 3). Significant different letters above the vertical bars indicate difference (* *P* < 0.05).

To investigate the potential role of *EsLHR* in ovarian maturation, the expression level of *EsLHR* transcripts in the ovaries, at various stages, was quantified by qPCR analysis. As shown in Figure 6, the relative expression level of *EsLHR* mRNA in Pvt, Lvt, and GVBD stages were significantly higher than that of Mvt and Evt stages (*P* < 0.05). The highest and the lowest expression were found in Lvt, and Evt, respectively (Figure 6). 

### 2.5. Induced Expression of EsLHR mRNA by the Crab GnRH Homolog

**Figure 7:**
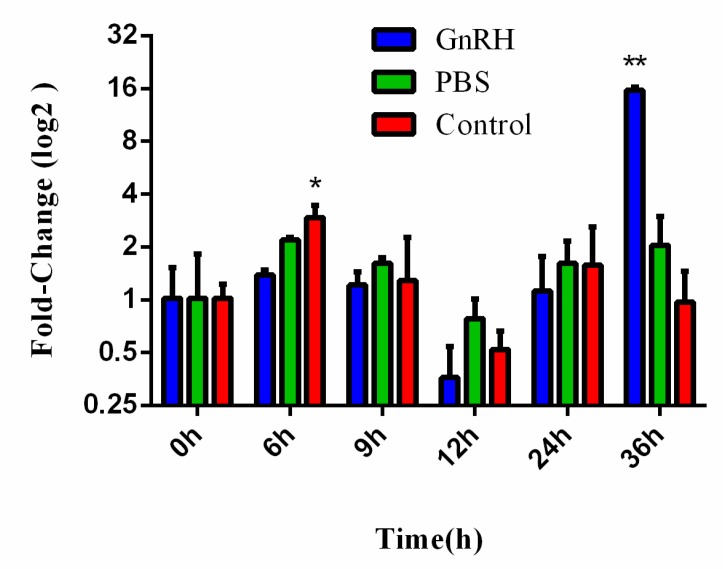
Induced expression of *EsLHR* mRNA by in vivo injection of the crab GnRH homolog. The blank control did not receive any treatment. The *β-actin* RNA was used as an internal control and the relative expression level of *EsLHR* was obtained relative to *β-actin* expression. The bars represent the mean ± SD (*n* = 3). * represents significant difference (*P* < 0.05), and ** represents highly significant difference (*P* < 0.01).

To further investigate whether the expression of *EsLHR* is regulated by GnRH, we performed in vivo injection experiment using the crab GnRH homolog. In ovary, the expression of *EsLHR* mRNA, in response to GnRH homolog induction, was examined by qPCR at 0 h, 6 h, 12 h, 24 h, and 36 h post injection. As shown in Figure 7, the relative expression level of *EsLHR* in GnRH group began to rise significantly at 36 h post injection (*P* < 0.01), whereas no significant change in PBS-injected group and blank control group. This data indicated that GnRH homolog can induce *EsLHR* mRNA expression.

## 3. Materials and Methods

### 3.1. Ethics Statement

The mitten crab *E. sinensis* is not an endangered or protected species, and permission to perform experiments involving this species is not required in China.

### 3.2. Animals and Tissue Sampling

Adult crabs were collected from a local aquaculture farm in Chongming District, Shanghai. All the crabs were acclimated in a fresh water circulation system aquarium, supplying sufficient dissolved oxygen for recovery. At the end of seven-day feeding, various tissues, including the brain, ovary, testis, eyestalk, heart, gill, muscle, thoracic ganglion and hepatopancreas, were dissected for subsequent RNA extraction. Additionally, ovarian tissues were fixed in 4% paraformaldehyde solution overnight at 4 °C for histological observation and in situ hybridization. The developmental stages of ovaries were classified according to previously published criteria [23]. 

### 3.3. GnRH Homolog Injection Experiments

The individuals were divided into three groups: (1) PBS group, 25 individuals were intraperitoneal injected with 100 μL PBS. (2) GnRH group, 25 individuals were intraperitoneal, injected with 100 μL GnRH solution, at the concentration of 500 ng GnRH/g body weight (BW). (3) Blank control group, 25 individuals did not receive any treatment. Ovarian tissues from each group were sampled and frozen by liquid nitrogen in 0 h, 6 h, 9 h, 12 h, 24 h, and 36 h postinjection, and the developmental state of ovarian tissues were all in late vitellogenic stage (Lvt) during the GnRH injection experiments.

### 3.4. Cloning of the Full-Length EsLHR Complementary DNA (cDNA)

Total RNA was extracted from the tissues using RNAiso Plus reagent (Takara, Japan), and cDNA was synthesized with SMARTer RACE (Rapid Amplification of cDNA Ends) cDNA amplification Kit (Clontech, Kusatsu, Japan) according to the manufacturer’s instructions. The full-length cDNA of *EsLHR* was obtained by 5′-RACE and 3′-RACE with gene-specific primers (Table 1). RACE-PCR was performed in a 50 μL mix containing 10 μL of 5× PCR buffer, 31.75 μL of PCR-grade water, 1.25 U of Takara Ex Taq® HS (Takara), 0.5 μL 5′-RACE or 3′-RACE gene-specific primer (10 μM), 5 μL Universal Primer A Mix (10×) and 2.5 μL of RACE-Ready cDNA, under the following cycle profile: 94 °C for 30 s followed by 35 cycles of 94 °C for 5 s, 55 °C for 30 s, and 72 °C for 1 min. The PCR products were ligated into pGEM®-T Easy vector (Promega, Madison, WI, USA), transformed into competent Escherichia coli DH5α cells, plated on a LB-agar Petri dish and incubated overnight at 37 °C. Positive clones containing the insert with expected size were identified by colony PCR. Eight of the positive clones were picked up and sequenced on an ABI PRISM3730 Automated Sequencer using BigDye terminator v3.1 (Applied Biosystems, Waltham, MA, USA).

### 3.5. Tissue Distribution of EsLHR Gene

Various tissues were collected from three adult individuals. Total RNA from the tissues was treated with DNase I (Promega) and submitted to reverse transcription (RT). The gene-specific primers used in PCR are shown in Table 1. The reaction conditions were 95 °C for 5 min, followed by 30 cycles of 94 °C for 30 s, 60 °C for 30 s, 72 °C for 1 min. Meanwhile, β-actin was amplified as an internal control using a pair of primers (Table 1). The RT-PCR products were separated by electrophoresis on a 2.0% agarose gel and verified by sequencing.

### 3.6. Probe Preparation and in situ Hybridization

A fragment of the cDNA (393 bp) of *EsLHR* was cloned and ligated into pGEMT-T Easy vector (Promega), and then the recombinant plasmid was linearized with Spe I (Takara). DIG-labeled probes of anti-sense and sense were synthesized by in vitro transcription, with a DIG RNA Labeling Kit (Roche Diagnostics, Mannheim, Germany), using the SP6, and T7 RNA polymerases (Takara), respectively. The ISH of ovarian tissue was carried out as described previously [24] with minor changes. Briefly, all tissue sections (~6 μm) were dehydrated and deparaffinized with xylene three times, for 5 min each times. After rinsing, all sections were treated with 3–5 μg·mL^−1^ proteinase K for 10 min at 37 °C. Subsequently, the slides were hybridized with DIG-labeled antisense or sense RNA probes two hours at 52 °C. After serial washing, the DIG was visualized using colorimetric substrates NBT/BCIP (Roche) according to the manufacturer’s instructions. For routine histological observation, the sections were stained with hematoxylin and eosin.

### 3.7. Real-Time qPCR Analysis

About 200 ng of total RNA from each sample was reverse-transcribed with iScript™ cDNA Synthesis Kit (Bio-Rad, Massachusetts, CA, USA) according to the manufacturer’s protocol. Melting curve analysis was performed to select the optimum primer pairs (Table 1). The resultant cDNAs were PCR-amplified in a volume of 10 ml that consisted of RT reaction, 16 Ex Taq buffer, 0.2 mM of each dNTP, 0.5 mM of each primer, and 0.25 U TaKaRa Ex TaqH Hot Start Version (Takara, Tokyo, Japan). The amplification reaction procedure was performed as follows: 94 °C for 3 min, followed by 25 cycles of 94 °C for 30 s, 57°C for 30 s, and 72 °C for 1 min. Real-time PCR was carried out using a Bio-Rad CFX96 Real-Time PCR Detection System (Bio-Rad) in a 20 μL reaction mix containing 1 μL cDNA template, 4 μL 5× iScript reaction mix (Bio-Rad), 0.5 μL of each primer (10 μM) and 14 μL nuclease-free water. The qPCR cycling conditions were as follows: 95 °C for 30 s, 40 cycles of 95 °C for 5 s and 60 °C for 30 s, followed by dissociation curve analysis at 95 °C for 15 s, 60 °C for 1 min and 95 °C for 15 s to verify the amplification of a single product. A reaction without cDNA was used as the negative control. The housekeeping gene β-actin was amplified as an internal reference. The expression levels were calculated using the 2^−△△*C*T^ method [25]. Each sample was run in triplicate. The relative expression of fold change was measured by the ratio of the target gene to that of internal reference gene within the same sample after log2 transformation. Data were presented as the mean ± standard error (SE). The mean difference was determined by one-way ANOVA analysis followed by t test or multiple range t test using SPSS statistical software (version 20.0) and a *P* value <0.05 was considered to be significantly different.

### 3.8. Bioinformatics Analysis

Nucleotide and amino acid sequences were identified using the BLAST program (http://blast.ncbi.nlm.nih.gov/Blast.cgi). The open reading frame (ORF) of *EsLHR* was determined by the ORF Finder (http://www.ncbi.nlm.nih.gov/projects/gorf/). The cleavage site for the putative signal peptide was predicted using online website (TMPRED, https://embnet.vital-it.ch/software/TMPRED_form.html). The deduced amino acid sequences of EsLHR were aligned with the amino acid sequence of LHRs, and related GPCRs, using the ClustalW program (ClustalW2, EMBL, Heidelberg, Germany). The representative sequences of EsLHR homolog were obtained from the GenBank database for phylogenetic analysis. A phylogenetic tree was constructed based on the deduced full-length amino acid sequence alignments by the Neighbor-Joining (NJ) algorithm method embedded in the MEGA software 5.0 (Aubencheul-au-bac, France). The bootstrap test was employed based on 10,000 pseudo-replications to assess the reliability of the phylogenetic tree.

## 4. Discussion

To the best of our knowledge, this study represents the first report on the identification of a *LHR* gene in decapods crustacean. The mitten crab *EsLHR,* encoding a protein, contained typical features of the known glycoprotein receptor LHR, including ECD, TMD, and ICD (Figure 1). Seven exons were detected in *EsLHR* genomic sequence. The seven-TMDs of EsLHR were coded by four exons, which is most similar to the wheat stem sawfly *Cehus cinctus* (Figure 2). In invertebrates, the genomic structures of *LHR* genes were quite different among species. For example, seven-TMDs were encoded by one exon in the horseshoe crab *Limulus polyphemus* and the scorpion *Centryroides sculpturatus*, whereas the TMDs were encoded with the last two exons in the flour beetle *Tribolium castaneum*. However, the genomic sequences of most vertebrates *LHRs* gene contain 11 exons, in which the last exon encodes for TMDs (Figure 2) [26]. These data indicated that the genomic structure of *LHR* gene was not fully conserved between the invertebrates and vertebrates, and the multiple exons, coding the transmembrane domains, could represent an ancestral LHR form. 

As an important signal transduction receptor for regulating gonad development, *LHR* mRNA was detected at the highest level in gonad of many fish species, such as the yellow catfish (*Pelteobagrus fulvidraco*), goldfish (*Lubricogobius exiguus*), mummichog (*Fundulus heteroclitus*) and sea bass (*Dicentrarchus labrax*) [12,27,28,29]. But, in African catfish (*Clarias gariepinus*) and *Cynoglossus semilaevis* [19,30], the expression of *LHR* was not the highest in the gonads. Our tissue expression studies revealed that *EsLHR* were expressed at the highest level in the ovary, suggesting an essential role for *EsLHR* in the reproduction of *E. sinensis*. It is has demonstrated that LH, coupled with its receptor LHR, plays a key role in controlling ovarian development in fish, especially in the final oocyte maturation. Generally, the LH-mediated control of ovarian maturation requires the timely and quantitative expression of the *LHR* in the ovary. [31,32]. Hence, we investigated the expression profiles of crab *EsLHR* during ovarian development. The qPCR result showed that *EsLHR* abundances in Lvt and GVBD were significantly higher than in Evt and Mvt stages (Figure 6). Similarly, a higher expression of the *LHR* mRNA was detected in a late stage of follicular development in bovine [33]. In zebrafish, the expression of *LHR* was hardly detected in the immature ovary, but initiated at the beginning of vitellogenesis and reached peak level at the full-grown oocyte stage [11]. These results indicated that, like vertebrate *LHR*, *EsLHR* may have implications in the final meiotic maturation of oocytes in *E. sinensis*. In additional, a higher expression of *EsLHR* mRNA was also detected in Pvt. This result was supported by subsequent ISH analysis. Strong mRNA hybrid-signal was mainly visualized in the ooplasm of Pvt oocytes (Figure 5B), suggesting that *EsLHR* could also have a potential role in Pvt stage besides in the final oocyte maturation. However, ISH analysis showed that the hybridization signal was undetectable in oocytes at Lvt and GVBD stages (Figure 5N). The inconsistent results between ISH and qPCR were also achieved in the characterization of other function genes [5,22,34]. The paradoxical for those data may result from the large amounts of yolk granules in the fully-grown oocyte at Lvt, which could inhibit probes from binding their target transcripts in ISH analysis. Also, the enlargement of oocytes seem to dilute the concentration of *EsLHR* mRNA [35].

In vertebrates, the synthesis and release of LH is stimulated by GnRH and gonad maturation is mediated via GnRH signaling pathway [36]. In decapod species, GnRH-like peptide of crab *Portunus pelagicus* was detected by using anti-GnRH antibody [37,38] and GnRH analogue can promote the ovarian maturation and spawning in giant freshwater prawn, *Macrobrachium rosenbergii* [39]. LH immune-signal was also detected in the brain of the mud crab *Scylla serrate* and the swimming crab *Portunus trituberculatus* by vertebrate LH antiserum [40,41]. In vivo injection of ovine LH can stimulating ovaries maturation of the sand shrimp *Crangon, crangon* [42]. In our previous study, a gonadotropin-releasing hormone (GnRH)-like peptide was partially isolated from the brain extract of Chinese mitten crab *E. sinensis* [43]. Synthesized the crab GnRH homolog peptide can induce GVBD of oocyte [44]. GnRH receptor (GnRHR) homolog was cloned and its corresponding GnRH signal pathway was revealed by transcriptomic analysis [22]. To investigate whether the crab EsLHR is involved in the GnRH signaling pathway, the expression of *EsLHR* mRNA was investigated by in vivo injection of the crab GnRH homolog. The relative expression level of *EsLHR* mRNA increased significantly at 36 h post injection (Figure 7), strongly suggesting that the expression *EsLHR* is regulated by GnRH homolg. 

In conclusion, this study presents the first report of the cloning and characterization of the *EsLHR* gene in *E. sinensis*. Significantly, the high expression of *EsLHR* in the ovaries at Pvt, Lvt, and GVBD stage suggested that *EsLHR* could have implications in both early ovarian development and the final meiotic maturation of GVBD oocytes. The crab GnRH homolog can induced *EsLHR* expression, indicating that EsLHR is involved in regulation of oocyte maturation through GnRH signaling pathway.

## Figures and Tables

**Table 1:** Primers used in this study.

Primers	sequence (5′ → 3′)	Usage
LHR-F1	ATGTCCTCTGGAAGTGCAAAGAT	RT-PCR
LHR-R1	TTACTGCTTGGAGAAGAAGTCTTTG	
LHR-F2	GGTGGCAGGTCTTCCGCTTAT	3′-RACE
LHR-R2	GCCCGTCTCAGGTTTGGTT	5′-RACE
LHR-F3	GGTGGCAGGTCTTCCGCTTAT	cloning for ISH probes
LHR-R3	GCCCGTCTCAGGTTTGGTT	
LHR-F4	TGGCAGGTCTTCCGCTTATT	qPCR
LHR-R4	GCCCTTGTACTTCATCGCTC	
β-actin F	CCTCACCCTCAAATACCCCAT	RT-PCR and qPCR
β-actin R	GGGGTGTTGAAGGTCTCGGA	
